# Gene Expression Profiling of Vasoregression in the Retina—Involvement of Microglial Cells

**DOI:** 10.1371/journal.pone.0016865

**Published:** 2011-02-17

**Authors:** Yuxi Feng, Yumei Wang, Li Li, Liang Wu, Sigrid Hoffmann, Norbert Gretz, Hans-Peter Hammes

**Affiliations:** 1 5^th^ Medical Department, Medical Faculty Mannheim, University of Heidelberg, Mannheim, Germany; 2 Medical Research Center, Medical Faculty Mannheim, University of Heidelberg, Mannheim, Germany; Universidade de Sao Paulo, Brazil

## Abstract

Vasoregression is a hallmark of vascular eye diseases but the mechanisms involved are still largely unknown. We have recently characterized a rat ciliopathy model which develops primary photoreceptor degeneration and secondary vasoregression. To improve the understanding of secondary vasoregression in retinal neurodegeneration, we used microarray techniques to compare gene expression profiles in this new model before and after retinal vasoregression. Differential gene expression was validated by quantitative RT-PCR, Western blot and immunofluorescence. Of the 157 genes regulated more than twofold, the MHC class II invariant chain CD74 yielded the strongest upregulation, and was allocated to activated microglial cells close to the vessels undergoing vasoregression. Pathway clustering identified genes of the immune system including inflammatory signaling, and components of the complement cascade upregulated during vasoregression. Together, our data suggest that microglial cells involved in retinal immune response participate in the initiation of vasoregression in the retina.

## Introduction

Regression of the matured retinal vasculature in adults is often initiated by pericyte loss and subsequent degeneration of endothelial cells [1], [2]. Extended vasoregression can result in hypoxia, and the dense retinal vascular network adapts to the high metabolic demands of neurons which are in close proximity to each other. Neurodegeneration such as retinitis pigmentosa leads to secondary vascular attenuation, leakage and functional limitations [3], [4]. Some investigations also suggest that damage of retinal glial cells (astrocytes, Müller cells and microglia) precedes vascular impairment [2], [5]. Microglial activation has been found in a variety of retinal pathologies such as photoreceptor degeneration, diabetes and ischemia- reperfusion [6], [7], [8]. However, the link between microglial activation and vasoregression is unclear.

The processes involved in the maturing vessels, comprise recruitment signals for pericytes, organization of basement membrane components, and secretion of inhibitors of (metallo-) proteases [9]. Consequently, regression of mature vessels requires the erroneous abrogation of survival promoting signals, and the aberrant activation of matrix-degrading proteases, among others. For example, in the diabetic retina, dropout of pericytes is conceived as a primary step in the reactivation of the endothelium, reducing endothelial protection [10]. The progressive independence of vessels for survival signals is one important characteristic of mature vessels. If VEGF is conditionally reduced in retinal tissue during postnatal development, it results in a loose capillary network with numerous regressive capillary profiles. However, when VEGF is inhibited during adulthood, there is no induction of vasoregression [11].

Impaired capillary formation is observed when neurodegeneration starts before the completion and maturation of the retinal network in animal models [12]. This contrasts with our observations in a novel model of adult retinal vasoregression. We recently characterized this transgenic rat displaying neuronal and vascular degeneration, and discovered that vasoregression ensues after the retinal capillary system had fully developed, preceded by neurodegeneration [13]. In this model, dropout of vascular pericytes, and capillary occlusions became evident after the second month of life. Thus, a precise image of the temporal and structural evolution of retinal vascular degeneration exists.

Despite an extensive vasoregression, hypoxia and instructive VEGF regulations were absent in this model, while some neurotrophins were upregulated, suggesting that a. the response to injury was probably inadequate, and b. other mechanisms are involved in the extensive vasoregressive process.

Therefore, in order to identify genes and signaling pathways involved in retinal vasoregression in this rat model, we performed a microarray analysis before and after the initiation of vasoregression. We observed a pronounced implication of components of the immune and the complement system, and identified CD74 specifically upregulated in perivascular microglia of the deep capillary in which the primary vascular lesion occurs.

## Methods

### Animals

All experiments in this study were performed in accordance with the ARVO statement for the use of animals ophthalmic and vision research, and the regional animal ethics committee. This study was approved by the ethics committee Regierungspräsidium Karlsruhe, approval ID: 35-9185.81/G-93/05. Male homozygous PKD-2-247 rats (TGR) and male Sprague-Dawley (SD) rats were used as controls. Generation and genotyping of the transgenic rats were described previously [14]. The rats were held in a 12 hours light and dark cycle with free access to food and drinking water. At 1 and 3 months of age, SD and TGR rats were anesthetized, and after sacrifice the eyes were immediately frozen for later preparation of total RNA, retina homogenate and whole mount retinal immunofluorescence staining.

### RNA isolation

Retinal total RNA was individually extracted using Trizol reagent (Invitrogen, Germany) according to the manufacturer's protocol. The quality and purity of RNA were controlled spectrophotometrically and estimated by electrophoresis on a 1% agarose gel.

### Microarrays and pathway analysis

cDNA and cRNA synthesis, and hybridization to arrays of type Rae230_2 from Affymetrix (Santa Clara, CA, USA) were performed according to the recommendations of the manufacturer. Three arrays were applied for each combination of rat transgene (TGR/SD) and age (1-month and 3-month). A total of 12 arrays were hybridized. The dependency of gene expression on rat transgene or age was analyzed using JMP Genomics (SAS Institute, Cary, NC, USA). Signals were first log transformed and quantile normalized, before being subjected to mixed model ANOVA, by which rat transgene and age as well as probe were considered as fixed effects and array-id as random. An ORA approach using Fisher's exact test was taken to identify pathways listed in Kyoto Encyclopedia of Genes and Genomes that are likely to be affected by differential gene expression. Additionally, gene set enrichment analysis (GSEA, version 2.0) and Ingenuity Systems software (Ingenuity Systems, Redwood City, CA, USA) were applied to reveal biological pathways modulated by rat transgene or age. Genes were ranked according to their expression levels. All Gene Ontology terms were examined using 1000 rounds of permutation of gene sets. MIAME compliant microarray data were submitted to Gene Expression Omnibus (GEO), sample number GSE20967.

### Quantitative real time PCR

For quantitative real time PCR, retinal total RNA was isolated using Trizol according to the manufacturer's protocol. The cDNA synthesis with removal of genomic DNA was performed using the QuantiTect^®^ Reverse Transcription kit (Qiagen GmbH, Hilden, Germany). TaqMan 2xPCR master Mix (Applied Biosystems, Weiterstadt, Germany) was applied for real time PCR. A volume of 20 µl of amplification reactions contained 0.4 µl cDNA, 1x Master Mix, 0.9 µM each primers and 0.25 µM probe. The samples were amplified using an ABI 7000 Real Time PCR System (Applied Biosystems, Darmstdt, Germany). Thermal cycling was carried out for 2 min at 50°C, then, for 10 min at 95°C, followed by 40 cycles of 15 sec at 95° and 1 min at 60°C. All primers and MGB probes labelled with FAM for amplification were purchased from Applied Biosystems: A2M, Rn 01459605_m1; B2M, Rn 00560865_m1; CD74, Rn 00565062_m1; CEBPB, Rn 00824635_s1; CFB, Rn 01526084_g1; CTSS, Rn 00569036_m1; RT1-DA, Rn 01427980_m1; Serping1, Rn 01485600_m1; C1qa, Rn 01519903_m1; TNFRSF1A, Rn 01492348_m1; IL-1b, Rn 00580432_m1; b-Actin, Rn 00667869_m1. Expression of genes was analysed by the 2^−ΔΔCT^ method using β-actin as a reference gene.

### Western blot

To assess protein expression of genes of interest, retinal proteins were individually extracted from TGR and SD rats of 1 and 3 months of age. The retinas were homogenized in 0.1% SDS buffer containing 125 mM NaCl, 10 mM EDTA, 25 mM Hepes, 10 mM Na3VO4, 0.5% deoxycholic acid, 0.1% SDS, 1% Triton X-100 with Complete™ protease inhibitor cocktail (Roche Diagnostic GmbH, Mannheim, Germany). The lysate was centrifuged at 10,000 rpm for 10 min. The supernatant was then collected and the protein concentration was determined with the Bradford protein assay according the manufacturer's protocol. 20–40 µg proteins were separated on 10–20% SDS gels under reducing conditions. Furthermore, the proteins were then transferred onto PVDF membranes and the membranes were blocked with 5% bovine serum albumin or 5% non-fat dry milk in Tris buffered with Tween-20 for 1 h to reduce non-specific binding. Then, the Blot was incubated with primary antibody over night at 4°C. After washing, they were further incubated with HRP-conjugated secondary antibody for 2 hours at room temperature. Finally, the signals were detected with enhance chemiluminescence (ECL) and exposed to X-film.

### Retinal Immunofluorescence

For whole mount retinal immunofluorescence staining, eyes were fixed in 4% paraformaldehyde overnight. After dissection retinas were washed in PBS and incubated in permeabilization and blocking buffer containing 1%BSA and 0.5% Triton for 1 hour. For detecting microglia activation, mouse anti CD11b (1∶100, AbD Serotec, Düsseldorf, Germany) or rabbit anti rat Iba1 (1∶50 DAKO, Hamburg, Germany), rabbit anti CD74 (Santa Cruz, Heidelberg, Germany) and Lectin labelled with biotin (1∶100, Sigma, Munich, Germany) were used. The corresponding secondary antibodies used were goat anti mouse IgG Alexa 488 (1∶200, Invitrogen, Karlsruhe, Germany), swine anti rabbit labelled with Tritc (1∶20, DAKO, Hamburg, Germany) and streptavidin labelled with Alexa 633 (1∶200, Invitrogen, Karlsruhe, Germany) for CD11b, CD74 and Lectin. For determination of CD74 expression in pericytes, rabbit anti NG2 (1∶200, Chemicon, Germany), goat anti CD74 (1∶50, Santa Cruz, Heidelberg, Germany) and Lectin labelled with biotin (1∶100, Sigma, Munich, Germany) were used. The corresponding secondary antibodies used were swine anti rabbit-TRITC (DAKO, Hamburg, Germany, 1∶20), donkey anti goat FITC (Acris, 1∶100) and streptavidin labelled with Alexa 633 (1∶200, Invitrogen, Karlsruhe, Germany) for CD11b, CD74 and Lectin, respectively. After washing in PBS, the retinas were flat mounted in 50% glycerol and photos were taken with a confocal microscope (Leica TCS SP2 Confocal Microscope, Leica, Wetzlar, Germany).

### Statistical analysis

Statistical analysis for microarray data was summarized above. Data of transcriptional expression are presented with mean and standard error. Analysis of variance (ANOVA) with Bonferroni post-test was performed to determine the significance between the groups. A p value less than 0.05 was considered as statistically significant.

## Results

### Genes and pathways involved in the development of vasoregression

**Figure 1:**
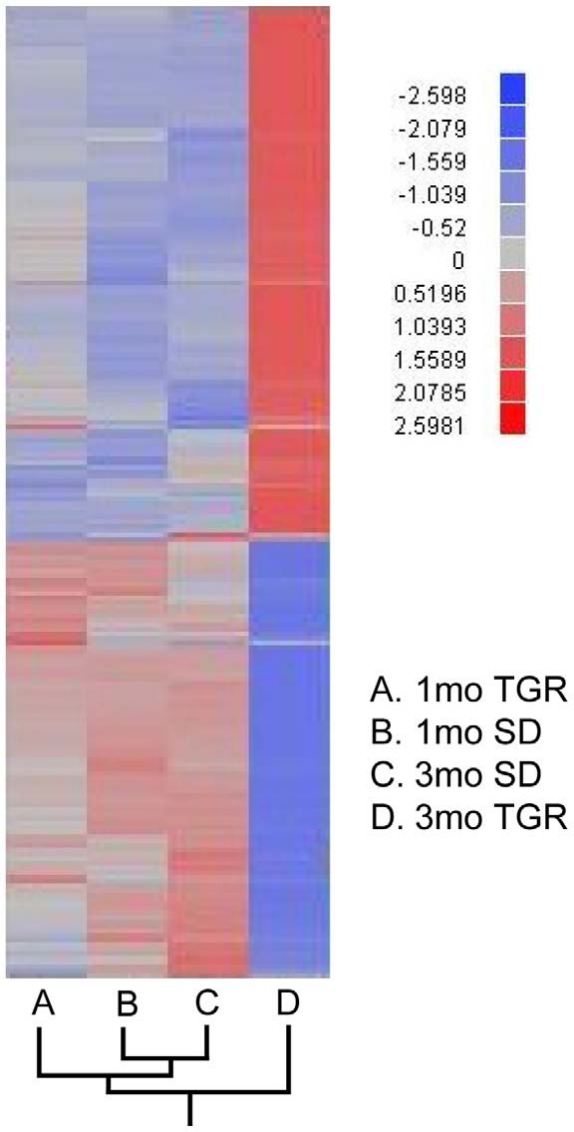
Hierarchical clustering analysis of gene profiling in SD and TGR rats at 1- and 3-month. Only genes with negative log10 P>10 are shown on the clustering diagram. The diagram shows that TGR 1-month, SD 1- and 3-month have similar gene expression profiling whereas 3-month TGR differs markedly from these three groups. Red indicates high and blue low expression of the single gene compared with the mean. The fold changes in the bar are represented in log_2_X. A: 1-month TGR; B: 1-month SD; C: 3-month SD; D: 3-month TGR.

**Figure 2:**
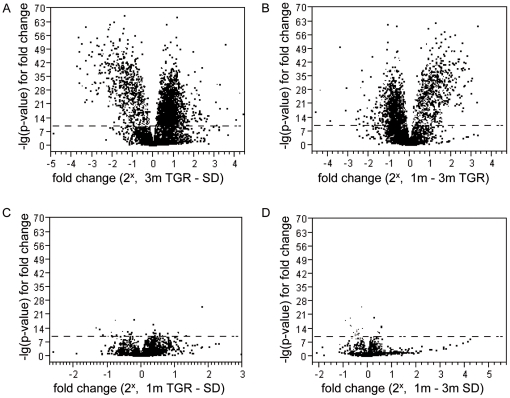
Volcano blots of significance against the fold change of expression of SD and TGR retinas at 1- and 3-month. The diagram shows that there are numerous genes in 3-month TGR up and down regulated compared with 3-month SD (A) and 1-month TGR (B). Few genes were differentially expressed among 1-month TGR and SD rats, 1- and 3-month SD rats. Horizontal reference line indicates negative log10 P>10 on the clustering diagram.

To identify genes and pathways involved in the development of vasoregression in TGR retinas, gene expression profiling was assessed in TGR and control rats at 1 and 3 months, i.e. before and after initiation of vasoregression, respectively. The Affymetrix GeneChip^®^ for rat expression array 230 2.0 was used. The array comprised of more than 31,000 probe sets for analysing over 30,000 transcripts and variants from over 28,000 rat genes. 3267 genes were significantly upregulated while 2924 genes were significantly downregulated when significance level (p-value) of less than 0.001 was defined as cutoff between TGR and SD rats. Moreover, hierarchical clustering of rat groups was performed based on differential gene expression (Figure 1). Genes of SD rats at 1 and 3 months exhibited similar expression patterns, and had a high comparability to the gene expression patterns of TGR rats at 1 month of age. 3-month old TGR rats demonstrated major changes in gene expression (Figure 2).

Next, we focused on genes which were differentially expressed in 3-month TGR over time (i.e. compared with 1-month TGR) and compared with controls (i.e. 3-month SD rats). As displayed in the volcano plot, a number of genes in 3-month TGR retinas exhibited significant expression changes when compared with 1-month TGR, and with 3-month SD rats. 374 genes were >2^+1^ or <2^−1^ significantly regulated during development of vasoregression (Table S1). Of these genes, 157 genes in 3-month TGR were more than 2-fold upregulated whereas 216 genes in this group were more than 50% downregulated in comparison with the two other groups. CD74 (invariant polypeptide of major histocompatibility complex, class II antigen-associated), Lgals3 (Lectin-galactose binding-soluble 3), and Serping1 (serine peptidase inhibitor-clade G, member 1) were the genes with the strongest upregulation in the 3-month TGR while Slc31a2 (solute carrier family 31-member 2), Hk2 (Hexokinase 2), Pax4 (paired box gene 4) and Rdh12 (retinol dehydrogenase 12) were ∼90% down-regulated. In contrast, there were only few genes which were regulated between TGR and SD retinas at 1 month, and in SD retinas between 1 and 3 months (Figure 2).

**Figure 3:**
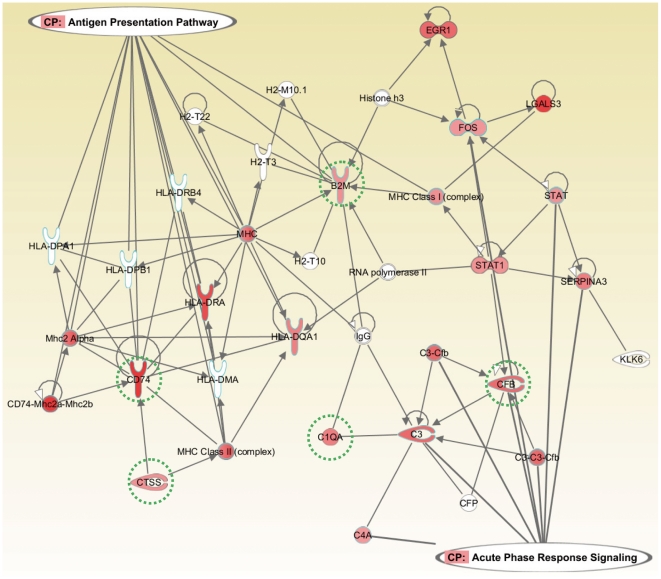
Antigen presentation and complement component response signaling predicted by Ingenuity Pathway Analysis in the TGR. All genes whose mRNA expression levels were differentially regulated in the TGR at 3 months are highlighted in red. Genes validated by real-time PCR are marked by green dotted circles. Symbols depict different gene functions. The upward facing fork shape represents transmembrane receptor, the right facing forceps shape represents peptidase, the dumbell shape represents transcription regulator.

Additionally, to examine the potential biological relevance of the transcriptome response during vasoregression, differentially expressed genes were classified according to their biological processes defined in Gene Ontology (GO) and Kyoto Encyclopedia of Genes and Genomes (KEGG). Functional analysis using GO revealed that a number of specific pathways were actively involved in the development of vasoregression in TGR rats at 3 months. Fourty pathways in GO were significantly upregulated during vasoregression in 3-month TGR compared with 1-month TGR and 3-month SD retinas. On the other hand, a total of five pathways were upregulated in KEGG. Furthermore, genes involved in the immune system including inflammatory and complementary response, cell signalling via receptors, especially protein tyrosine kinase activity, exhibited strong correlation with vasoregression. Some genes were shared by different pathways, such as CD74 antigen in the immune effector process, in antigen processing and presentation, in inflammatory response and in cytokine binding; CCAAT/enhancer binding protein beta (CEBPB) was shared in pathways of the immune response, in defence response and in inflammatory response. Figure 3 depicts the two central pathways, i.e. the antigen presentation pathways, and the acute response pathway, and their relationships.

Transmembrane receptor tyrosine kinases, which are relevant for vascular development and remodelling such as tyrosine kinase with immunoglobulin-like and EGF-like domains 1 (Tie1), VEGF receptor-2 (KDR), VEGF receptor-1 (FLT1), platelet-derived growth factor receptor alpha (PDGFRA), Epidermal-Growth-Factor-Receptor (EGFR) and fibroblast growth factor receptors (FGFR) were also found to be significantly changed (data not shown).

As internal validation, and based on previous results, we found GFAP 9 fold upregulated in the 3-month TGR rats compared with their age-matched controls in the array analysis [13].

### Validation of microarray data by quantitative real-time PCR

**Figure 4:**
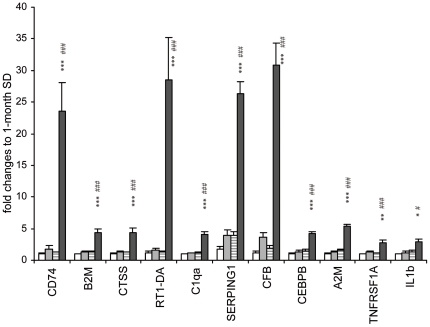
Confirmation of gene expression profiles in SD and TGR retinas at 1- and 3-month. Transcriptional analysis of genes was performed by quantitative real-time PCR in rats of 1-month SD (white bars), 1-month TGR (gray bars), 3-month SD (striped bars) and 3-month TGR (black bars). The values at 1-month are standardized to 1. *p<0.05, **p<0.01, ***p<0.001: 3-month TGR compared with 1-month TGR. #p<0.05, ###p<0.001: 3-month TGR compared with 3-month SD.

To validate the differential transcriptional gene expression observed on microarray analysis, we carried out TaqMan probe based on quantitative real-time PCR with independently retrieved TGR and SD retinas at 1 and 3 months of age. As shown in Figure 4, mRNA levels of CD74 were high in TGR retinas at 3 months, whereas expression of CD74 was low in TGR retinas at 1 month and SD retinas at 1 and 3 months. Similarly, A2M (alpha-2-macroglobulin), B2M (beta-2-macroglobulin), CEBPB, CFB (complement factor B), CTSS (cathapsin S), RT1-DA (RT1 class II, locus Da), SERPING1 (serine/cysteine peptidase inhibitor, clade G, member 1), C1qa (complement component 1, q subcomponent, alpha polypeptide) and TNFRSF1A (Tumor necrosis factor receptor superfamily member 1A) were expressed significantly higher in 3-month TGR retinas than in other three groups. These ten genes retrieved from microarray analysis were fully confirmed in quantitative real time PCR. Expression of CD74 detected was 19-fold higher in 3-month TGR retinas compared with age-matched SD rats in real time PCR analysis. Upregulated transcripts of B2M (3-fold), RT1-DA (23-fold) and CTSS (3-fold) which participate in antigen processing and presentation were confirmed by quantitative real-time PCR. Furthermore, complement regulation mediated by CFB (16-fold), SERPING1 (7-fold), and C1qa (3-fold) was also confirmed by real time PCR. Upregulation in inflammatory responses via A2M (4-fold), CEBPB (3-fold), TNFRSF1A (3-fold) and IL-1b (2-fold) were also confirmed. Thus, the expression levels of regulated genes from microarray analysis were fully confirmed by quantitative real time PCR.

### CD-74 expressing microglia localizes to retinal vasoregression

**Figure 5:**
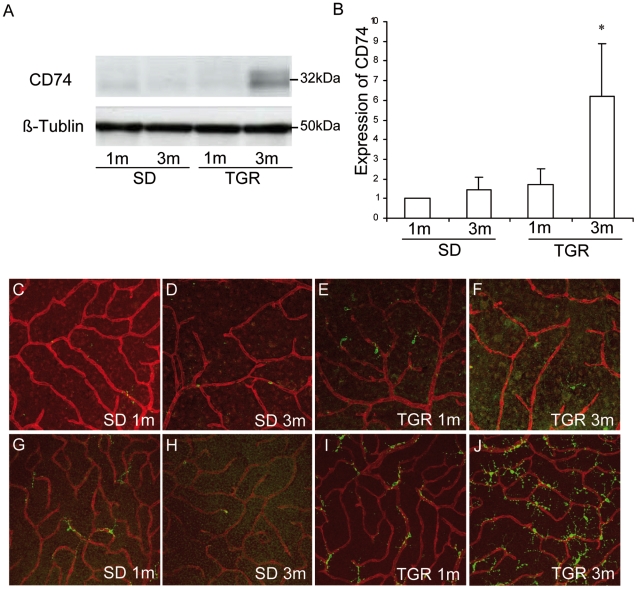
Expression of CD74 in SD and TGR retinas at 1- and 3 months. **A.** Translational analysis of CD74 by Western blot. **B.** quantitative analysis of translational CD74 expression. The values at 1-month are standardized to 1. C: expression and localization of CD74 in the retina. Vessels are visualized by staining with Lectin (red), and CD74 positive cells are displayed in green. Few CD74 positive cells are seen in SD in the superficial and deep capillary layers. A slight increase in number of CD74 positive cells is found in TGR in the superficial capillary layer at 1 month. The numbers of CD74 positive cells are raised in the deep capillary layer in 3-month TGR compared with other three groups at all time points observed. C–F: the superficial capillary layer; G–J: the deep capillary layer. 1 m: 1-month retina. 3 m: 3-month retina. *p<0.05: 3-month TGR compared with other three groups.

To investigate translational expression levels of CD74 obtained on the microarray, we performed western blot analysis with retinal lysates from TGR and SD rats at 1 and 3 months. Weak CD74 expression was found in the retinas of 1- and 3-month SD and 1-month TGR rats. A significant overexpression of CD74 protein was detected in 3-month TGR retinas compared with other three groups (Figure 5A and B).

**Figure 6:**
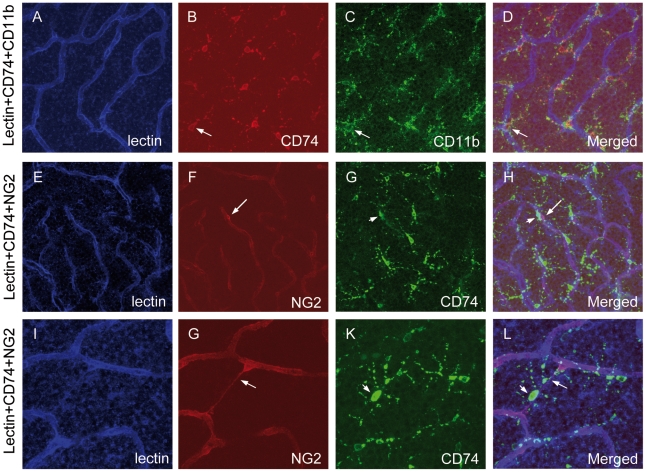
Activation of microglial cells in TGR retinas. Vessels and microglial cells are visualized by staining with Lectin (blue) and CD11b (green), respectively. CD74 (red) is expressed in microglial cells (A–D). Furthermore, CD74 (green) is not detected in pericytes as demonstrated by the staining with NG2 (red), a pericyte marker (E–H). Moreover, microglial cells expressing CD74 were found to be close to regressive capillary (I–L). Arrows in A–D: colocalization of CD74 and CD11b; arrows in E–H: pericyte; arrowheads in E–H: CD74 positive microglial cell. Arrows in I–L: regressive vessel.

Next, we identified by immunofluorescence that CD74 was expressed in cells close to both, the superficial and deep capillary layers in SD rats. In contrast, CD74 positive cells were located predominantly around the deep capillary in TGR rats of one month of age. The strongest and most obvious accumulation of CD74-positive cells was found in TGR rats of three months in the vicinity of the deep capillary layers. CD74 positive cells were either localized on or closed to the vessels or in the inter-vascular spaces with prolonged end feet spanning vessels. The morphology of these cells identified them as microglial cells which were further confirmed by co-immunostaining with microglial marker CD11b and Iba-1 (Figure 6A, B, C, D and Figure S1). CD74 was expressed predominantly in the membrane of cell soma and dot-like in the processes of microglial cells, whereas CD11b labelled strongly all processes of microglial cells. Even though CD74-positive microglial cells were localised close to vessels and some even seemed morphologiocally like pericytes, immunofluorescence staining revealed no colocalisation between CD74 positive microglia cells and pericytes labelled with pericyte-specific marker NG2 (Figure 6E, F, G, H). 66% CD74 positive microglial cells were close to the capillaries. Close correlation between CD74 positive microglial cells and regressive vessels was noted in 3-month TGR retinas (Figure 6I, J, K, L).

We analysed expression of macrophage-inhibiting factor (MIF) known as ligand for CD74 in various tissues and diseases. We compared protein expression in the four groups analyzed and did not find any upregulated expression in the TGR groups (data not shown). Thus, MIF as ligand for CD74 seems not to be relevant in the neurodegenerative retina.

## Discussion

In our model of ciliopathy-associated neurodegeneration, we identified CD74 and other components of the immunity system, expressed in microglia, as mediators of secondary vasoregression. Genes involved in the immune response including inflammatory/complementary pathways and in tyrosine kinase pathway were strongly regulated. Our findings suggest that CD74 positive microglial cells may have a function during the development of vasoregression in the retina.

The first novel finding in our study suggests that immune system including antigen processing and presentation, inflammatory and complementary response plays a predominant role during vasoregression in TGR rat. Activation of these pathways was verified by upregulation of CD74/B2M/RT1-DA/CTSS, A2M/CEBPB/TNFRSF1A/IL-1b and CFB/SERPING1/C1qa, respectively. The representative molecule from antigen processing and presentation as well as inflammatory pathways during vasoregression is CD74. CD74 is the invariant chain directing the trafficking of MHC class II molecules in antigen presenting cells (APCs) [15]. Previous studies have shown that CD74 is associated with cancer and inflammatory disease, such as Helicobacter pylori infection, bladder inflammation, atherosclerosis and renal injury [16], [17]. Moreover, upregulation of members of the innate immunity and of the complement cascade has been uniformly observed in several other animal models of post-developmental retinal injury (Table S2). For example, the model of acute IOP elevation resembles the PKD model in that some components of the innate immunity and the complement system were comparatively regulated. However, the involvement of the microglia was not established. Of note, by comparing our data with data of other models, the link between CD74 expression on microglia and the close proximity to vasoregression was not made.

Signal transduction studies indicated that CD74 binds MIF, and in cooperation with CD44, mediates a variety of ERK1/2 and src tyrosine kinase-based cellular functions such as endothelial proliferation, regulation of apoptosis, regulation of arachidonic and prostaglandin metabolism [18].

Apart from APCs, cancer cells and podocytes, expression of CD74 is increased in smooth muscle cells undergoing inflammation and in microglia in neurodegenerative disorder such as Alzheimer disease [19], [20]. We found that CD74 is expressed in microglial cells during vasoregression in TGR retinas. Of note, our model represents a ciliopathy-based photoreceptor degeneration with secondary vascular defects. Thus, our data which show similarities to gene expression profiles of other inflammatory and neurodegenerative diseases suggest that CD74 may represent an important link between inflammatory stimuli and vasoregression.

Interestingly, we observed a strong upregulation of CD74 preceding and accompanying neurodegeneration and vasoregression in the absence of major changes in retinal MIF levels. Thus, CD74 mediated tissue damage may indicate a predominant immune cell mediated antigen processing and presentation process. This is supported by the finding that various genes of the complement system such as C1qa, CFB and Serping1 are highly upregulated in the TGR model.

Antigen binding of C1qa binding is the first step in activating the classical complement pathway, whereas CFB and Serping1 are regulators in the alternative and classical complement activation pathways [21].

Recent genetic studies show that polymorphisms in complement factor B and H (CFH) are important for the development of age-related macular degeneration (AMD) and its vascular pathology. A study from von Leithner et al. demonstrated that CFH is essential in the maintenance of endothelial function for normal retina perfusion [22]. Deficiency in CFB causes an autoimmune response directly in the endothelium of intra-retinal vascular network. Retinal vessels in CFH knockout mice are progressively overloaded with C3 and C3b, leading to vasoconstriction and occlusion. While CFB promotes complement activation after binding to C3b, CFH suppresses the activation upon binding to C3b. CFH and CFB are closely associated with C3 and the formation of C5 convertase. Together, complement activation can cause vascular damage in a variety of pathological settings. The multiple interplay of components of the classical and alternative complement pathway in association with the activated immune system provide a possible mechanistic link between photoreceptor-degeneration induced upregulation of CD74 activation in microglia and secondary vasoregression, but the causal relationship between complement activation and vasoregression needs further substantiation.

The proximity of CD74 positive microglia to the capillary network which is primarily affected in the TGR suggests that these cells are somehow involved in the onset of vasoregression. Mature vessels are largely independent of growth survival signals than immature vessels. Thus, adult vasoregression in the presence of upregulated survival factors is extraordinary. However, the role of microglia in the destruction of retinal vessels is suggestive given the evidence, e.g. from the RCS rat and other models in which photoreceptor degeneration, microglia activation, and vessel regression have been demonstrated [6], [23], [24].

However, neither model has provided evidence that CD74 and the complement system are central mediators. From our data, we postulate that defects in cilia of the photoreceptor cells lead to apoptosis, hypothetically releasing peptides and cell debris that activate microglial cells.

Our model represents specific features which are considered a general responsive phenotype of the retina. In a recent review by Xu et al., the aging retina shows “para-inflammation” as characteristic multicellular response pattern, including low level apoptosis, complement activation and inflammatory cytokine reaction, and the activation of microglia as an important effector cell. By comparison, the TGR model shares some of these important characteristics, however, also with some singularities, such as the extensive vasoregression [25].

Adult vasoregression may be promoted when pericytes are lost yielding reduced endothelial protection. The TGR has reduced pericyte coverage over time for unknown reasons, rendering it more susceptible to vasoregression in analogy to mice with heterozygous PDGF-B deficiency. Activated microglia is known to secrete inflammatory cytokines such TNF-alpha and IL-1b which can activate and subsequently damage endothelial cells [26]. Further studies inhibiting inflammatory signalling in this model are required to delineate the importance of these secondary tissue responses.

Of note, some of the genes upregulated in the TGR are consistent with genes expressed in Müller glia isolated from 6 months diabetic SD retinas [27]. These include CD74, RT1-Ba, C1s, A2M, Timp1 and CEBP. Many genes associated with immune/inflammatory and acute-phase response produced by Müller cells are also strongly regulated in our TGR rat model. Interestingly, CD74 was not found in astrocytes or Müller cells in the TGR. Whether this is due to differences in species or in inducing mechanisms needs further investigations. Together, the data indicate that vasoregression in TGR retinas shares similarities with molecular changes induced by chronic hyperglycemia.

As we determined gene expression before and after vessel regression, the involvement of specific tyrosine kinases in relation to vasoregression is not surprising. Given the progressive glial response to the neurodegeneration with concomitant activation and expression of neurotrophin which share survival activities of vessels, the responsive upregulation of the respective tyrosine kinase pathways appears as an epiphenomenon of the ongoing damage.

In summary, our study on analysis of gene expression profiling identified pathways involved in immune/inflammatory/complement system that act as regulators in the development of vasoregression in the degenerating retina. CD74 and activated microglial cells might be a novel target for interfering with retinal vasoregression.

## Supporting Information

Figure S1 **Colocalization of Iba 1 and CD74 in the TGR retina.** A: Iba 1; B: CD74; C: Lectin; D: merged image of A–C.
(PPTX)

Table S1 Genes regulated in 3-month TGR retinas compared with 1-month TGR and 3-month SD retinas. Genes more than 2fold upregulated or more than 30% downregulated are included in the table.
(PDF)

Table S2 Comparison of gene expression in the TGR model with other animal models.
(PDF)
