# Supplementary material for: Gene Expression Profiling of Vasoregression in the Retina—Involvement of Microglial Cells
Source: PLoS One. 2011 Feb 17;6(2):e16865. doi: 10.1371/journal.pone.0016865 (PMC3040753; doi:10.1371/journal.pone.0016865)
Supplement: Table S1 — Genes regulated in 3-month TGR retinas compared with 1-month TGR and 3-month SD retinas. Genes more than 2fold upregulated or more than 30% downregulated are included in the table. (PDF) [file pone.0016865.s002.pdf]

feng et al supplementary table 1

Probe_Set_	Gene_Title	Gene_Symbol	pkd 1m - sd 1m (fold)	sd 3m - sd 1m (fold)	pkd 3m - pkd 1m (fold)	pkd 3m - sd 3m (fold)
1367679_a	CD74 antigen (invariant polypeptide of major histocon	Cd74	1,19	1,32	24,03	21,64
1386879_a	lectin, galactose binding, soluble 3	Lgals3	0,99	1,07	20,54	18,99
1372254_a	serine (or cysteine) peptidase inhibitor, clade G, mem	Serping1	2,30	2,06	14,89	16,64
1377659_a	myeloid leukemia factor 1 (predicted)	Mlf1_predicted	3,16	1,40	5,41	12,25
1387893_a	complement component 1, s subcomponent	C1s	1,39	1,25	8,77	9,75
1388155_a	keratin 18	Krt18	1,22	0,49	3,82	9,47
1368000_a	complement component 3	C3	1,34	1,64	11,51	9,39
1368321_a	early growth response 1	Egr1	0,59	0,21	3,31	9,35
1368353_a	glial fibrillary acidic protein	Gfap	4,08	1,41	3,22	9,29
1389470_a	complement component 2 /// complement component	Bat4 /// C2 /// C4a ///	1,05	1,23	10,48	8,88
1387276_a	activity and neurotransmitter-induced early gene prote	Ania4	2,42	1,16	4,10	8,55
1367712_a	tissue inhibitor of metalloproteinase 1	Timp1	1,58	1,09	5,74	8,33
1387995_a	interferon induced transmembrane protein 3	Ifitm3	1,61	0,85	3,63	6,85
1376652_a	complement component 1, q subcomponent, alpha pc	C1qa	1,26	1,09	5,67	6,54
1368224_a	serine (or cysteine) peptidase inhibitor, clade A, meml	Serpina3n	1,02	1,04	6,63	6,50
1368419_a	ceruloplasmin	Cp	1,87	1,39	4,76	6,39
1370822_a	RT1 class II, locus Ba	RT1-Ba	0,98	0,97	6,12	6,22
1387770_a	interferon, alpha-inducible protein 27-like	Ifi27l	1,00	0,89	5,48	6,17
1368840_a	transmembrane protein 176B	Tmem176b	1,24	0,75	3,42	5,67
1392171_a	chitinase 3-like 1	Chi3l1	3,49	1,65	2,66	5,61
1374236_a	leucine-rich repeat LGI family, member 2 (predicted)	Lgi2_predicted	1,03	0,97	4,98	5,34
1387343_a	CCAAT/enhancer binding protein (C/EBP), delta	Cebpd	2,17	1,25	2,95	5,11
1386913_a	podoplanin	Pdpn	1,68	0,89	2,71	5,09
1370913_a	radical S-adenosyl methionine domain containing 2	Rsad2	1,13	0,91	4,11	5,07
1370973_a	sodium channel, voltage-gated, type VII, alpha	Scn7a	1,07	1,05	4,94	5,03
1387005_a	cathepsin S	Ctss	1,10	1,18	5,31	4,93
1387946_a	lectin, galactoside-binding, soluble, 3 binding protein	Lgals3bp	1,02	0,91	4,28	4,78
1387088_a	galanin	Gal	1,08	1,06	4,61	4,71
1370964_a	argininosuccinate synthetase 1	Ass1	1,67	1,17	3,17	4,52
1367794_a	alpha-2-macroglobulin	A2m	1,91	1,52	3,54	4,45
1370892_a	complement component 4a /// advanced glycosylation	Ager /// Agpat1 /// Btr	1,02	1,20	5,16	4,41
1367595_s	beta-2 microglobulin	B2m	1,16	1,01	3,72	4,30
1373204_a	transmembrane protein 176A	Tmem176a	1,47	1,10	3,13	4,19
1387599_a	NAD(P)H dehydrogenase, quinone 1	Nqo1	1,11	1,06	3,99	4,17
1387583_a	cytochrome P450, family 26, subfamily A, polypeptide	Cyp26a1	1,15	0,97	3,43	4,06
1379717_a	similar to ATP-binding cassette, sub-family G (WHITE	LOC360997	1,19	1,07	3,56	3,95
1372757_a	signal transducer and activator of transcription 1	Stat1	1,30	1,09	3,26	3,91
1367974_a	annexin A3	Anxa3	1,10	1,01	3,50	3,83
1367581_a	secreted phosphoprotein 1	Spp1	0,77	0,91	4,51	3,83

1368658_a	ciliary neurotrophic factor	Cntf	1,34	1,14	3,23	3,79
1371942_a	glutathione S-transferase theta 1 /// glutathione S-tran	Gstt1 /// Gstt3	1,03	0,87	3,11	3,70
1370182_a	protein tyrosine phosphatase, receptor type, N polype	Ptprn2	1,26	1,03	2,70	3,32
1367784_a	clusterin	Clu	1,42	1,01	2,30	3,25
1377804_a	protogenin homolog (Gallus gallus)	Prtg	1,61	1,03	2,04	3,19
1388164_a	RT1 class Ib, locus S3	RT1-S3	0,74	1,01	4,32	3,18
1389179_a	cell death-inducing DNA fragmentation factor, alpha s	Cidea_predicted	1,04	0,81	2,49	3,18
1370709_a	leucine rich repeat containing 15	Lrrc15	1,19	1,06	2,77	3,12
1398362_a	Notch gene homolog 2 (Drosophila)	Notch2	1,14	0,88	2,40	3,11
1386940_a	tissue inhibitor of metalloproteinase 2	Timp2	1,21	0,98	2,49	3,07
1388784_a	colony stimulating factor 1 receptor	Csf1r	1,01	0,79	2,38	3,07
1370237_a	hydroxyacyl-Coenzyme A dehydrogenase	Hadh	0,98	0,74	2,29	3,04
1387223_a	aminoadipate aminotransferase	Aadat	1,23	1,68	4,16	3,04
1395473_a	guanine nucleotide binding protein, beta 3	Gnb3	1,77	1,19	2,04	3,03
1376427_a	glycine decarboxylase (predicted)	Gldc_predicted	1,40	1,19	2,50	2,96
1390383_a	adipose differentiation related protein	Adfp	1,13	0,97	2,54	2,95
1373847_a	transmembrane 4 superfamily member 1 (predicted)	Tm4sf1_predicted	1,24	0,89	2,12	2,94
1388753_a	sulfatase 2	Sulf2	1,30	1,20	2,69	2,92
1388213_a	RT1 class Ib gene, H2-TL-like, grc region (N3) /// RT1	Dhx16 /// Ier3 /// Mrps	0,79	0,93	3,41	2,90
1389360_a	FXYD domain-containing ion transport regulator 3	Fxyd3	1,24	1,15	2,69	2,89
1371447_a	placenta-specific 8 (predicted)	Plac8_predicted	1,02	0,93	2,63	2,87
1371194_a	tumor necrosis factor alpha induced protein 6	Tnfaip6	1,06	0,91	2,45	2,86
1370043_a	activated leukocyte cell adhesion molecule	Alcam	1,28	1,03	2,27	2,83
1372828_a	methionine sulfoxide reductase B2	Msrb2	1,14	0,81	2,01	2,82
1383546_a	glycerophosphodiester phosphodiesterase domain co	Gdpd2_predicted	1,19	0,96	2,27	2,81
1376845_a	putative ISG12(b) protein	isg12(b)	0,99	1,00	2,84	2,81
1376702_a	megalencephalic leukoencephalopathy with subcortica	Mlc1_predicted	1,42	1,25	2,47	2,80
1389034_a	ubiquitin specific peptidase 18	Usp18	0,97	0,95	2,73	2,80
1369537_a	melanin-concentrating hormone receptor 1	Mchr1	1,07	1,07	2,78	2,78
1373152_a	protease, serine, 23	Prss23	1,21	0,88	2,03	2,77
1371536_a	calcium regulated heat stable protein 1	Carhsp1	1,18	0,98	2,29	2,77
1370247_a	peripheral myelin protein 22	Pmp22	1,20	1,01	2,31	2,76
1370870_a	malic enzyme 1, NADP(+)-dependent, cytosolic	Me1	1,28	1,18	2,55	2,76
1375030_a	UDP-Gal:betaGlcNAc beta 1,3-galactosyltransferase,	B3galt5_predicted	1,06	1,09	2,83	2,75
1367896_a	carbonic anhydrase 3	Car3	0,93	0,94	2,78	2,75
1388480_a	glycolipid transfer protein (predicted)	Gltp_predicted	1,16	0,94	2,22	2,74
1379891_a	scotin	MGC94600	1,19	0,90	2,07	2,74
1369959_a	zinc finger protein 36, C3H type-like 1	Zfp36l1	1,23	0,90	2,01	2,74
1369751_a	thyrotropin releasing hormone receptor	Trhr	1,02	1,23	3,30	2,74
1390437_a	sema domain, seven thrombospondin repeats (type 1	Sema5a_predicted	1,12	0,87	2,13	2,73

1374474_a	copine VIII (predicted)	Cpne8_predicted	1,13	0,92	2,22	2,72
1367604_a	cysteine-rich protein 2	Crip2	1,28	1,10	2,33	2,70
1370383_s	transporter 1, ATP-binding cassette, sub-family B (MD	Btnl3 /// Btnl4 /// Btnl5	0,98	0,98	2,70	2,70
1391593_a	Ras association (RalGDS/AF-6) domain family 4	Rassf4	1,09	0,97	2,37	2,69
1387209_a	SEC16 homolog B (S. cerevisiae)	Sec16b	1,27	1,10	2,31	2,66
1383050_a	proline-rich polypeptide 6	Prr6	1,19	1,04	2,32	2,66
1368075_a	lysosomal acid lipase A	Lipa	1,23	1,15	2,48	2,66
1368806_a	selenoprotein P, plasma, 1	Sepp1	1,13	0,89	2,09	2,65
1383564_a	interferon regulatory factor 7	Irf7	0,91	1,08	3,15	2,64
1398623_a	cholinergic receptor, nicotinic, beta polypeptide 4	Chrnb4	1,20	1,13	2,48	2,63
1378209_a	serine/arginine-rich protein specific kinase 3	Srpk3	1,68	1,33	2,09	2,63
1398435_a	solute carrier family 22 (organic cation transporter), m	Slc22a15_predicted	1,16	1,21	2,72	2,61
1372805_a	LOC363015 (predicted)	RGD1310444_predic	1,13	0,90	2,07	2,60
1367800_a	plasminogen activator, tissue	Plat	1,18	0,92	2,01	2,60
1367631_a	connective tissue growth factor	Ctgf	1,54	1,36	2,30	2,60
1373750_a	leprecan-like 2 (predicted)	Leprel2_predicted	1,79	1,48	2,15	2,60
1374417_a	similar to nuclear receptor binding protein	LOC680451	1,61	1,36	2,20	2,60
1388064_a	solute carrier family 1 (glial high affinity glutamate tra	Slc1a3	1,19	1,09	2,37	2,58
1368134_a	interleukin 4 receptor, alpha	Il4ra	1,03	0,99	2,47	2,57
1386862_a	annexin A5	Anxa5	1,04	0,88	2,15	2,56
1375640_a	FK506 binding protein 9	Fkbp9	1,35	1,08	2,05	2,56
1374310_a	protein phosphatase 1J	Ppm1j	1,08	1,09	2,57	2,55
1375637_a	similar to RIKEN cDNA 1110003E01	RGD1311122	1,01	0,85	2,16	2,55
1377086_a	C1q and tumor necrosis factor related protein 3 (predi	C1qtnf3_predicted	1,17	0,98	2,12	2,54
1386899_a	cathepsin H	Ctsh	1,16	1,00	2,19	2,54
1367691_a	protein kinase C, delta binding protein	Prkcdbp	1,02	0,86	2,13	2,53
1397537_a	similar to Discs large homolog 5 (Placenta and prosta	LOC363434 /// LOC5	0,95	0,80	2,10	2,51
1378430_a	monooxygenase, DBH-like 1	Moxd1	1,30	1,12	2,16	2,51
1370215_a	complement component 1, q subcomponent, beta poly	C1qb	1,10	0,96	2,19	2,50
1372297_a	glutathione S-transferase, alpha 4	Gsta4	1,06	0,89	2,11	2,50
1371537_a	UDP-Gal:betaGlcNAc beta 1,4-galactosyltransferase,	B4galt5_predicted	1,21	1,11	2,27	2,49
1381374_a	leucine-rich repeat LGI family, member 4	Lgi4	1,45	1,34	2,29	2,48
1390738_a	bone marrow stromal cell antigen 2	Bst2	0,92	1,05	2,83	2,48
1398863_a	guanine nucleotide binding protein, beta 2	Gnb2	1,16	0,98	2,08	2,47
1372455_a	tetraspanin 12	Tspan12	1,18	1,08	2,25	2,47
1389600_a	hypothetical protein LOC363306 /// similar to Discs la	LOC363306 /// LOC3	1,01	0,85	2,06	2,44
1368332_a	guanylate nucleotide binding protein 2	Gbp2	1,07	1,05	2,35	2,41
1390050_a	similar to Golgi phosphoprotein 2 (Golgi membrane p	LOC680692 /// LOC6	1,15	1,13	2,35	2,41
1387370_a	tropomodulin 1	Tmod1	1,38	1,18	2,07	2,41
1374718_a	deltex 3-like (Drosophila)	Dtx3l	1,02	1,03	2,39	2,38

1368059_a	crystallin, mu	Crym	1,32	1,24	2,23	2,37
1371696_a	G protein-coupled receptor 56	Gpr56	1,12	1,04	2,20	2,37
1377046_a	ankyrin repeat domain 6	Ankrd6	1,31	1,19	2,14	2,36
1396860_s	zinc finger protein 423	Zfp423	1,08	0,97	2,11	2,35
1369672_a	arachidonate 5-lipoxygenase activating protein	Alox5ap	1,04	0,93	2,09	2,35
1395274_a	dystonin (predicted)	Dst_predicted	1,29	1,15	2,08	2,34
1389815_a	protein phosphatase 1, regulatory (inhibitor) subunit 1	Ppp1r14b	1,23	1,18	2,24	2,34
1383241_a	complement component 1, r subcomponent	C1r	1,13	1,05	2,16	2,33
1372752_a	tetraspanin 4	Tspan4	1,18	1,18	2,31	2,32
1377076_a	sialic acid acetylesterase (predicted)	Siae_predicted	1,07	0,96	2,07	2,30
1390707_a	regulator of G-protein signalling 10	Rgs10	1,12	1,01	2,07	2,28
1373240_a	dehydrogenase/reductase (SDR family) member 3	Dhrs3	0,83	0,96	2,65	2,28
1368869_a	A kinase (PRKA) anchor protein (gravin) 12	Akap12	1,17	1,12	2,18	2,27
1370885_a	cathepsin Z	Ctsz	1,13	1,05	2,11	2,27
1390638_a	Eph receptor A4	Epha4	1,06	0,99	2,10	2,25
1367777_a	2,4-dienoyl CoA reductase 1, mitochondrial	Decr1	0,93	0,85	2,06	2,25
1390832_a	transmembrane and coiled coil domains 3	Tmcc3	1,08	1,02	2,10	2,23
1373037_a	ubiquitin-conjugating enzyme E2L 6	Ube2l6	0,91	0,99	2,44	2,23
1387283_a	myxovirus (influenza virus) resistance 2	Mx2	0,97	0,98	2,24	2,23
1376102_a	transmembrane BAX inhibitor motif containing 1	Tmbim1	1,10	1,06	2,13	2,22
1379275_a	sorting nexin 10	Snx10	0,89	1,01	2,53	2,21
1374752_a	MyoD family inhibitor domain containing (predicted)	Mdfic_predicted	0,86	0,87	2,24	2,21
1376693_a	similar to OEF2 (predicted)	RGD1563091_predic	1,06	1,06	2,18	2,19
1374425_a	transducin-like enhancer of split 1, homolog of Drosop	Tle1_predicted	1,02	1,03	2,19	2,18
1367786_a	proteasome (prosome, macropain) subunit, beta type	Psmb8	1,08	1,08	2,17	2,17
1369157_a	phosphodiesterase 3B, cGMP-inhibited	Pde3b	1,19	1,13	2,05	2,17
1385625_a	EGF-containing fibulin-like extracellular matrix protein	Efemp2	1,32	1,22	2,00	2,16
1373122_a	ajuba	Jub	1,02	0,99	2,08	2,16
1387369_a	exocyst complex component 6	Exoc6	1,26	1,29	2,20	2,14
1389653_a	protocadherin beta 9	Pcdhb9	1,13	1,08	2,04	2,14
1373025_a	complement component 1, q subcomponent, C chain	C1qc	1,09	1,06	2,07	2,14
1369964_a	coronin, actin binding protein 1A	Coro1a	1,07	1,05	2,06	2,11
1373891_a	zinc finger, CCHC domain containing 12	Zcchc12	1,01	1,02	2,13	2,10
1386718_a	aldo-keto reductase family 1, member C19	Akr1c19	1,07	1,25	2,42	2,08
1383662_a	hypothetical protein LOC500956	LOC500956	1,13	1,17	2,14	2,06
1374730_a	Tyro protein tyrosine kinase binding protein	Tyrobp	1,01	1,01	2,01	2,01
1369960_a	FXYD domain-containing ion transport regulator 1	Fxyd1	0,89	1,01	2,27	2,01
1372112_a	similar to 9230105E10Rik protein	RGD1304579	1,03	1,04	2,02	2,00
1374589_a	vezatin, adherens junctions transmembrane protein	Vezt	0,89	0,90	0,50	0,50
1369895_s	podocalyxin-like	Podxl	1,02	1,01	0,49	0,49

1370281_a	fatty acid binding protein 5, epidermal	Fabp5	0,93	0,66	0,35	0,49
1382616_a	glutaminase	Gls	1,11	0,98	0,43	0,49
1398361_a	Rho GTPase activating protein 10	Arhgap10	0,97	0,91	0,46	0,49
1381403_a	malic enzyme 2, NAD(+)-dependent, mitochondrial (pı	Me2_predicted	0,94	0,87	0,45	0,49
1368025_a	DNA-damage-inducible transcript 4	Ddit4	1,00	1,00	0,48	0,49
1369352_a	homeodomain interacting protein kinase 3	Hipk3	1,22	1,13	0,45	0,49
1367624_a	activating transcription factor 4	Atf4	0,92	0,95	0,50	0,48
1374472_a	vacuolar protein sorting 37 homolog A (S. cerevisiae)	Vps37a	1,07	1,03	0,46	0,48
1397838_a	RGD1564792 (predicted)	RGD1564792_predic	1,04	1,05	0,49	0,48
1386981_a	solute carrier family 16 (monocarboxylic acid transpor	Slc16a1	0,86	0,86	0,48	0,48
1367910_a	MAD homolog 4 (Drosophila)	Smad4	0,98	1,02	0,49	0,47
1381801_a	fascin homolog 2, actin-bundling protein, retinal (Stroı	Fscn2_predicted	1,34	1,27	0,45	0,47
1375987_a	ceramide kinase (predicted)	Cerk_predicted	1,08	0,78	0,33	0,47
1398427_a	Myocyte enhancer factor 2D	Mef2d	0,99	1,04	0,48	0,46
1369260_a	membrane protein, palmitoylated 4 (MAGUK p55 subf	Mpp4	0,95	0,85	0,41	0,46
1374205_a	purine rich element binding protein B	Purb	0,92	0,95	0,47	0,46
1374206_a	similar to hypothetical protein DKFZp434A1319 (predi	RGD1307357_predic	1,04	1,03	0,45	0,46
1371565_a	KTI12 homolog, chromatin associated (S. cerevisiae)	Kti12	1,11	1,19	0,49	0,46
1375085_a	solute carrier family 25, member 35	Slc25a35	1,08	0,94	0,40	0,46
1393017_a	rhophilin, Rho GTPase binding protein 1 (predicted)	Rhpn1_predicted	1,03	0,89	0,39	0,46
1373897_a	Lamin B1	Lmnb1	0,92	1,00	0,49	0,45
1382152_a	jumonji, AT rich interactive domain 1B (Rbp2 like)	Jarid1b	0,92	1,00	0,49	0,45
1390918_a	ADP-ribosylhydrolase like 1 /// GH regulated TBC prot	Adprhl1 /// Grtp1	1,08	1,08	0,45	0,45
1371923_a	lysophosphatidylcholine acyltransferase 1	Lpcat1	0,98	0,93	0,43	0,45
1390065_a	potassium channel, subfamily V, member 2 (predictec	Kcnv2_predicted	1,35	0,96	0,32	0,45
1376694_a	limb region 1-like homolog (mouse)	Lmbr1l	0,94	1,01	0,48	0,45
1383007_a	Bardet-Biedl syndrome 4 homolog (human) (predictec	Bbs4_predicted	0,96	0,82	0,38	0,45
1367767_a	3-hydroxy-3-methylglutaryl-Coenzyme A lyase	Hmgcl	0,80	0,89	0,50	0,44
1372016_a	growth arrest and DNA-damage-inducible 45 beta	Gadd45b	1,41	1,33	0,41	0,44
1367747_a	ADP-ribosylation factor-like 3	Arl3	0,88	0,97	0,49	0,44
1370912_a	heat shock 70kD protein 1B (mapped)	Hspa1b	1,01	0,98	0,43	0,44
1398280_a	interphotoreceptor matrix proteoglycan 1	Impg1	0,99	1,05	0,47	0,44
1378656_a	Bardet-Biedl syndrome 5	Bbs5	1,01	1,06	0,45	0,43
1384436_a	similar to Transmembrane protein 16B	LOC683001	0,99	0,88	0,38	0,43
1369431_a	UDP-N-acetyl-alpha-D-galactosamine:polypeptide N-a	Galnt7	0,75	0,86	0,49	0,43
1393573_a	phosphodiesterase 6B, cGMP, rod receptor, beta poly	Pde6b_predicted	0,90	1,01	0,48	0,43
1375517_a	tumor protein p53 inducible nuclear protein 2	Trp53inp2	0,96	1,04	0,46	0,43
1382105_a	guanine nucleotide binding protein (G protein), beta 5	Gnb5	0,90	0,98	0,46	0,42
1380619_a	similar to RIKEN cDNA 3110001I22 (predicted)	RGD1305537_predic	0,89	0,99	0,47	0,42
1369437_a	solute carrier organic anion transporter family, membe	Slco4a1	1,11	1,08	0,41	0,42

1388025_a	opsin 1 (cone pigments), short-wave-sensitive (color b	Opn1sw	0,87	0,49	0,23	0,42
1370258_a	basic leucine zipper and W2 domains 2	Bzw2	1,01	0,97	0,40	0,42
1384603_a	ATP-binding cassette, sub-family A (ABC1), member	Abca4_predicted	0,90	1,01	0,47	0,42
1392498_a	protein phosphatase 2, regulatory subunit B' gamma i	Ppp2r5c	0,86	0,94	0,46	0,42
1389146_a	family with sequence similarity 107, member B	Fam107b	0,95	1,04	0,45	0,41
1370828_a	zinc finger, DHHC domain containing 2	Zdhhc2	0,94	0,94	0,41	0,41
1388562_a	START domain containing 7 (predicted)	Stard7_predicted	0,90	1,07	0,49	0,41
1373666_a	Rap guanine nucleotide exchange factor (GEF) 5	Rapgef5	0,89	1,02	0,47	0,41
1382715_a	deleted in liver cancer 1	Dlc1	0,93	1,04	0,45	0,41
1373154_a	similar to mKIAA0998 protein (predicted)	RGD1563583_predic	0,92	0,93	0,41	0,41
1377721_a	Park2 co-regulated	Pacrg	0,92	1,06	0,47	0,41
1387166_a	aryl hydrocarbon receptor-interacting protein-like 1	Aipl1	0,98	1,00	0,41	0,41
1391749_a	similar to nucleoredoxin	LOC679020	1,03	0,97	0,38	0,40
1375677_a	transducer of ERBB2, 2	Tob2	1,02	1,04	0,41	0,40
1392425_x	LUC7-like (S. cerevisiae)	Luc7l	1,00	1,06	0,43	0,40
1373132_a	proline rich 14	Prr14	1,09	1,16	0,43	0,40
1398562_a	amyotrophic lateral sclerosis 2 (juvenile) chromosome	Als2cr4_predicted	0,93	0,91	0,39	0,40
1373103_a	metastasis-associated gene family, member 2	Mta2	0,98	1,12	0,45	0,40
1370376_a	cold shock domain protein A	Csda	0,96	0,91	0,37	0,40
1385848_s	tudor domain containing 7	Tdrd7	0,87	0,98	0,45	0,39
1389666_a	rod outer segment membrane protein 1	Rom1	0,94	0,94	0,40	0,39
1384220_a	tubulin-specific chaperone c (predicted)	Tbcc_predicted	0,99	0,95	0,38	0,39
1387152_a	nuclear receptor binding factor 2	Nrbf2	0,94	1,15	0,48	0,39
1398304_a	frizzled homolog 2 (Drosophila)	Fzd2	1,18	1,51	0,50	0,39
1373485_a	Similar to ring finger protein 122 homolog (predicted)	RGD1561238_predic	0,99	0,96	0,38	0,39
1389434_a	Bardet-Biedl syndrome 9	Bbs9	0,79	0,89	0,44	0,39
1375455_a	guanylate cyclase activator 1a (retina) (predicted)	Guca1a_predicted	0,87	1,10	0,49	0,39
1387703_a	ubiquitin specific peptidase 2	Usp2	0,76	0,91	0,46	0,39
1383390_a	family with sequence similarity 123A	Fam123a	0,88	0,90	0,39	0,38
1378246_a	PTC7 protein phosphatase homolog (S. cerevisiae)	Pptc7	0,85	1,10	0,50	0,38
1389485_a	transformed mouse 3T3 cell double minute 1	Mdm1	0,88	0,97	0,42	0,38
1394943_a	Similar to tyrosine kinase-associated leucine zipper pr	RGD1565584_predic	0,93	1,17	0,48	0,38
1388044_a	6-phosphofructo-2-kinase/fructose-2,6-biphosphatase	Pfkfb2	0,81	1,01	0,48	0,38
1388948_a	START domain containing 10	Stard10	0,85	1,05	0,47	0,38
1374454_a	protein-L-isoaspartate (D-aspartate) O-methyltransfer	Pcmtd2_predicted	0,83	0,90	0,41	0,38
1370101_a	cone-rod homeobox containing gene	Crx	0,91	0,95	0,39	0,38
1373384_a	Similar to Serine/threonine-protein phosphatase 2A 56	LOC691318	0,88	0,94	0,40	0,38
1370351_a	tudor domain containing 7	Tdrd7	0,90	0,99	0,41	0,38
1374534_a	ras homolog gene family, member T2	Rhot2	0,96	1,07	0,42	0,38
1368087_a	protein tyrosine phosphatase, non-receptor type 21	Ptpn21	0,90	1,13	0,47	0,38

1382220_a	insulin-like growth factor 2 mRNA binding protein 2	Igf2bp2	0,95	0,71	0,28	0,38
1371752_a	RAN guanine nucleotide release factor	Rangrf	0,95	0,95	0,37	0,37
1369360_a	guanylate cyclase 2e	Gucy2e	0,83	0,89	0,40	0,37
1368725_a	jagged 1	Jag1	0,85	1,09	0,48	0,37
1388373_a	adiponectin receptor 1	Adipor1	0,91	1,03	0,42	0,37
1378256_a	RNA polymerase II associated protein 1	Rpap1	0,76	0,97	0,48	0,37
1374428_a	kinesin family member 3B (predicted)	Kif3b_predicted	0,84	1,06	0,47	0,37
1367702_a	acyl-Coenzyme A dehydrogenase, medium chain	Acadm	0,73	0,92	0,47	0,37
1375073_a	similar to hypothetical protein MGC45873 (predicted)	RGD1310271_predic	0,96	1,23	0,47	0,37
1369821_a	cyclic nucleotide gated channel beta 1	Cngb1	0,88	0,94	0,39	0,37
1376057_a	Phosphodiesterase 8A	Pde8a	0,98	1,04	0,39	0,37
1380515_a	Bardet-Biedl syndrome 7	Bbs7	0,84	1,08	0,47	0,36
1390110_a	uridine-cytidine kinase 1-like 1	Uckl1	0,79	1,02	0,47	0,36
1393126_a	ubiquitin domain containing 1	Ubtd1	0,80	0,90	0,41	0,36
1391356_a	IWS1 homolog (S. cerevisiae)	Iws1	0,86	1,16	0,48	0,36
1370510_a	aryl hydrocarbon receptor nuclear translocator-like	Arntl	0,79	0,89	0,40	0,36
1369140_a	recoverin	Rcvrn	0,99	0,94	0,34	0,36
1388539_a	plakophilin 2	Pkp2	0,90	0,97	0,38	0,36
1368233_a	general transcription factor IIF, polypeptide 2	Gtf2f2	0,81	0,91	0,40	0,36
1377822_a	coiled-coil domain containing 64	Ccdc64	0,88	0,99	0,40	0,35
1368247_a	heat shock 70kD protein 1A /// heat shock 70kD protei	Hspa1a /// Hspa1b	1,08	0,95	0,31	0,35
1392970_a	transmembrane protein 108	Tmem108	0,97	1,03	0,37	0,35
1374768_a	coiled-coil domain containing 65	Ccdc65	0,76	0,98	0,45	0,35
1390334_a	similar to hypothetical protein DKFZp434I2117 (predic	RGD1308215_predic	0,97	0,94	0,34	0,35
1376048_a	guanylate cyclase activator 1B (predicted)	Guca1b_predicted	1,01	0,94	0,32	0,34
1389808_a	hypothetical protein LOC688274	LOC688274	0,95	1,18	0,43	0,34
1374929_a	coiled-coil domain containing 126	Ccdc126	0,80	1,10	0,47	0,34
1387452_a	nuclear transcription factor-Y beta	Nfyb	0,81	0,90	0,37	0,34
1389628_a	phospholipase C, delta 3 (predicted)	Plcd3_predicted	0,83	0,98	0,39	0,33
1398295_a	solute carrier family 29 (nucleoside transporters), mer	Slc29a1	0,89	0,96	0,36	0,33
1382320_a	LOC362793	RGD1307315	0,98	1,31	0,44	0,33
1373426_a	Mitogen activated protein kinase 1	Mapk1	0,80	0,82	0,34	0,33
1367676_a	high mobility group box 2	Hmgb2	0,82	0,95	0,38	0,33
1368242_a	potassium voltage gated channel, Shab-related subfai	Kcnb1	0,93	0,99	0,34	0,33
1392575_a	zinc finger protein 654	Zfp654	0,89	1,16	0,42	0,32
1370013_a	cyclic nucleotide gated channel alpha 1	Cnga1	0,92	1,00	0,35	0,32
1374333_a	similar to RIKEN cDNA 1110007C09 (predicted)	RGD1306058_predic	0,70	0,83	0,38	0,32
1379683_a	Similar to hypothetical protein MGC17839 (predicted)	RGD1565079_predic	0,86	1,11	0,41	0,32
1376118_a	OTU domain, ubiquitin aldehyde binding 2 (predicted)	Otub2_predicted	0,81	1,00	0,39	0,32
1372808_a	similar to Bifunctional methylenetetrahydrofolate dehy	LOC680308	0,73	1,02	0,44	0,32

1393098_a	Similar to Lethal giant larvae homolog 2 (predicted)	RGD1560307_predic	0,96	1,09	0,36	0,31
1393426_a	phosphodiesterase 6A, cGMP-specific, rod, alpha (pre	Pde6a_predicted	1,03	0,88	0,27	0,31
1371073_a	UDP-Gal:betaGlcNAc beta 1,4- galactosyltransferase,	B4galt1_predicted	1,13	1,09	0,30	0,31
1393684_a	helicase, lymphoid specific	Hells	1,01	1,20	0,37	0,31
1369693_a	solute carrier family 1 (glial high affinity glutamate tra	Slc1a2	1,07	0,99	0,29	0,31
1390338_a	similar to K04F10.2	LOC361646	1,00	1,10	0,34	0,31
1376017_a	similar to WD repeat domain 17	LOC361188 /// LOC3	0,80	1,01	0,39	0,31
1390221_a	PR domain containing 1, with ZNF domain (predicted)	Prdm1_predicted	0,98	1,11	0,35	0,31
1382551_a	intersectin 2	Itsn2	0,65	0,91	0,43	0,31
1394384_a	dopamine receptor D4	Drd4	0,95	1,26	0,40	0,30
1367732_a	guanine nucleotide binding protein, beta 1	Gnb1	0,93	0,96	0,31	0,30
1374563_a	similar to AMME syndrome candidate gene 1 protein I	RGD1561004_predic	0,87	0,96	0,33	0,30
1388023_a	calcium channel, voltage-dependent, beta 2 subunit	Cacnb2	0,89	1,05	0,34	0,29
1373091_a	pleiomorphic adenoma gene-like 2 (predicted)	Plagl2_predicted	0,92	1,00	0,31	0,29
1398604_a	oxysterol binding protein 2 (predicted)	Osbp2_predicted	0,87	0,97	0,32	0,29
1384089_a	RAB guanine nucleotide exchange factor (GEF) 1 (pre	Rabgef1_predicted	0,88	1,11	0,36	0,28
1398580_a	WD repeat domain 31	Wdr31	1,02	0,74	0,21	0,28
1368778_a	solute carrier family 6 (neurotransmitter transporter, ta	Slc6a6	0,74	0,97	0,37	0,28
1368139_s	alkaline phosphatase, liver/bone/kidney	Alpl	0,86	1,03	0,34	0,28
1388432_a	optineurin	Optn	0,77	0,94	0,34	0,28
1375407_a	Retinitis pigmentosa GTPase regulator interacting pro	Rpgrip1	0,67	1,03	0,43	0,28
1396038_a	retbindin	Rtbdn	0,88	1,11	0,35	0,28
1373816_a	adaptor-related protein complex 1, gamma 1 subunit	Ap1g1	0,65	0,98	0,42	0,28
1393936_a	serine/threonine kinase 22 substrate 1	Stk22s1	0,76	0,94	0,34	0,28
1390586_a	similar to Jumonji/ARID domain-containing protein 1C	RGD1560601_predic	0,77	1,07	0,38	0,27
1380997_a	cyclin J (predicted)	Ccnj_predicted	0,90	0,89	0,27	0,27
1381136_a	retinol binding protein 3, interstitial	Rbp3	0,79	0,90	0,31	0,27
1380824_a	hook homolog 3 (Drosophila)	Hook3	0,69	0,84	0,33	0,27
1373324_a	dual specificity phosphatase 14 (predicted) /// similar t	Dusp14_predicted ///	0,78	0,98	0,33	0,27
1373184_a	chondroitin sulfate N-acetylgalactosaminyltransferase	Csgalnact2	0,88	1,03	0,31	0,27
1385544_a	neural retina leucine zipper gene (predicted)	Nrl_predicted	0,80	1,05	0,34	0,26
1389741_a	LAG1 homolog, ceramide synthase 4	Lass4	0,87	0,92	0,28	0,26
1388363_a	hnRNP-associated with lethal yellow	Raly	0,72	0,96	0,34	0,26
1371767_a	SWI/SNF related, matrix associated, actin dependent	Smarcd1_predicted	0,75	0,97	0,33	0,25
1376867_a	galectin-related protein	Hspc159	0,91	0,94	0,26	0,25
1380383_a	ADP-ribosylation factor 4-like (predicted)	Arf4l_predicted	0,87	0,98	0,28	0,25
1393036_a	proline/serine-rich coiled-coil 1	Psrc1	1,11	1,06	0,24	0,25
1397854_a	D4, zinc and double PHD fingers, family 3 (predicted)	Dpf3_predicted	0,65	1,25	0,48	0,25
1382940_a	galactosidase, beta 1-like 2	Glb1l2	0,73	0,97	0,33	0,25
1378405_a	zinc finger protein 143	Zfp143	0,76	1,26	0,41	0,25

1369248_a	baculoviral IAP repeat-containing 4	Birc4	0,72	1,12	0,38	0,24
1384943_a	similar to DNA segment, Chr 16, ERATO Doi 472, exp	RGD1563888_predic	0,75	1,05	0,34	0,24
1378433_a	similar to RIKEN cDNA 5730557B15 (predicted)	RGD1564227_predic	0,87	0,97	0,27	0,24
1374375_a	similar to 2610034M16Rik protein (predicted)	RGD1560925_predic	0,69	1,19	0,41	0,24
1377250_a	similar to RIKEN cDNA A930008G19 (predicted)	RGD1310799_predic	0,72	0,92	0,31	0,24
1384112_a	5' nucleotidase, ecto	Nt5e	0,91	1,05	0,27	0,24
1370970_a	potassium inwardly-rectifying channel, subfamily J, m	Kcnj14	0,91	1,06	0,28	0,24
1368248_a	CDP-diacylglycerol synthase 1	Cds1	0,75	1,02	0,32	0,24
1372541_a	transmembrane protein 138	Tmem138	0,88	1,12	0,30	0,23
1384894_a	retinitis pigmentosa 1 homolog (human)	Rp1h	0,85	1,06	0,29	0,23
1390653_a	WD repeat domain 89	Wdr89	0,73	1,18	0,38	0,23
1367937_a	myo-inositol oxygenase	Miox	0,65	1,21	0,43	0,23
1373918_a	retinol dehydrogenase 11	Rdh11	0,83	0,94	0,26	0,23
1391022_a	laminin, beta 3	Lamb3	0,61	1,31	0,49	0,23
1372650_a	dynamin binding protein	Dnmbp	0,87	1,15	0,30	0,23
1367802_a	serum/glucocorticoid regulated kinase	Sgk	0,73	1,09	0,34	0,23
1380424_a	UBX domain containing 5	Ubxd5	0,91	1,31	0,32	0,22
1372824_a	pleckstrin homology domain containing, family F (with	Plekhf2_predicted	0,99	1,04	0,23	0,22
1380046_a	Hect domain and RLD 3 (predicted)	Herc3_predicted	0,69	0,91	0,29	0,22
1373587_a	guanine nucleotide binding protein, alpha transducing	Gnat1_predicted	0,96	0,95	0,22	0,22
1388501_a	hypothetical protein LOC686179	MGC125239	0,80	1,05	0,28	0,21
1383851_a	coiled-coil domain containing 96	Ccdc96	0,70	1,06	0,30	0,20
1370044_a	Fas apoptotic inhibitory molecule	Faim	0,69	0,86	0,25	0,20
1385472_a	zinc finger and BTB domain containing 8 (predicted)	Zbtb8_predicted	0,87	1,00	0,22	0,19
1383779_a	ribosomal RNA processing 1 homolog B (S. cerevisiae	Rrp1b	0,82	0,95	0,21	0,19
1389231_a	SH3-domain GRB2-like (endophilin) interacting protei	Sgip1	0,59	1,18	0,36	0,18
1387829_a	solute carrier family 24 (sodium/potassium/calcium ex	Slc24a1	0,81	0,96	0,21	0,18
1388913_a	phosphatidic acid phosphatase type 2c	Ppap2c	0,88	0,86	0,17	0,18
1395235_a	nucleoredoxin-like 1	Nxnl1	0,61	1,44	0,41	0,17
1384046_a	Mitochondrial fission regulator 1 (predicted)	Mtfr1_predicted	0,82	1,31	0,27	0,17
1395311_a	isoprenylcysteine carboxyl methyltransferase	Icmt	0,82	0,94	0,19	0,17
1374912_a	kinesin family member 2C	Kif2c	0,66	0,93	0,23	0,17
1374139_a	cerebellar degeneration-related 2	Cdr2	0,65	0,96	0,24	0,16
1384639_a	receptor accessory protein 6	Reep6	0,73	0,92	0,20	0,16
1370462_a	hyaluronan mediated motility receptor (RHAMM)	Hmmr	0,98	1,19	0,19	0,16
1370461_a	hyaluronan mediated motility receptor (RHAMM)	Hmmr	0,97	1,09	0,18	0,16
1385588_a	calcium binding protein 4 (predicted)	Cabp4_predicted	0,92	1,10	0,19	0,16
1375504_a	polymerase (DNA directed), gamma 2, accessory sub	Polg2_predicted	0,60	0,90	0,23	0,15
1376963_a	dual-specificity tyrosine-(Y)-phosphorylation regulatec	Dyrk2_predicted	0,92	1,04	0,17	0,15
1369160_a	solute carrier family 4, sodium bicarbonate cotranspoi	Slc4a7	0,62	0,84	0,20	0,15

1396569_a	1-acylglycerol-3-phosphate O-acyltransferase 3 (predi	Agpat3_predicted	0,74	0,84	0,16	0,14
1369625_a	aquaporin 1	Aqp1	0,65	0,84	0,16	0,13
1377351_a	sushi domain containing 3 (predicted)	Susd3_predicted	0,75	0,85	0,14	0,13
1371484_a	hypothetical protein LOC690349	LOC690349	0,72	0,87	0,15	0,12
1369257_a	G protein-coupled receptor kinase 1	Grk1	0,72	1,03	0,16	0,11
1369913_a	opsin 1 (cone pigments), medium-wave-sensitive (col	Opn1mw	0,71	1,02	0,15	0,11
1373410_a	myocyte enhancer factor 2C	Mef2c	0,64	1,59	0,26	0,10
1394972_a	TROVE domain family, member 2 (predicted)	Trove2_predicted	0,46	1,18	0,27	0,10
1382949_a	retinol dehydrogenase 12 (predicted)	Rdh12_predicted	0,82	0,81	0,10	0,10
1370140_a	paired box gene 4	Pax4	0,40	1,34	0,34	0,10
1392899_a	protein regulator of cytokinesis 1 (predicted)	Prc1_predicted	0,56	1,09	0,17	0,09
1383519_a	Hexokinase 2	Hk2	0,66	0,93	0,12	0,08
1377014_a	similar to hypothetical protein MGC42105 (predicted)	RGD1308116_predic	0,55	1,11	0,16	0,08
1373657_a	solute carrier family 31, member 2	Slc31a2	0,56	0,93	0,12	0,07
