# Supplementary material for: Gene Expression Profiling of Vasoregression in the Retina—Involvement of Microglial Cells
Source: PLoS One. 2011 Feb 17;6(2):e16865. doi: 10.1371/journal.pone.0016865 (PMC3040753; doi:10.1371/journal.pone.0016865)
Supplement: Table S2 — Comparison of gene expression in the TGR model with other animal models. (PDF) [file pone.0016865.s003.pdf]

Feng et al. Supplementary table 2

Comparison of gene expression in TGR model with other animal models

Disease	Animal model	genes	Function	Our data	References
Retinitis pigmentosa	*rd1* mice	Pde6b (↓, 0.17)<br>C1qa (↑, 1.60)<br>C1qb (↑, 3.0)<br>C1qc (↑, 1.8)<br>C4 (↑, 1.1)<br>Cebpd (↑, 3.2)<br>Pde6a (↓, 0.33)	visual transduction<br>Complementary response<br>Complementary response<br>Complementary response<br>Complementary response<br>Transcription factor<br>visual transduction	Pde6b (↓, 0.43)<br>C1qa (↑, 6.54)<br>C1qb (↑, 2.5)<br>C1qc (↑, 2.14)<br>C4 (↑, 8.88)<br>Cebpd (↑, 5.11)<br>Pde6a (↓, 0.31)	[1]<br><br><br><br><br><br>[2]
Autosomal recessive achromatopsia or early onset progressive cone dystrophy	*cpfl1* (cone photoreceptor function loss 1) mouse	A2m (↑ 1.501)<br>Cebpb (↑ 7.80)<br>C1qa (↑ 2.306)<br>C1qb (↑ 2.184)<br>C1qc (↑ 1.764)<br>C4 (↑ 5.257)<br>Serping1 (↑ 4.490)<br><br>Serpina3n (↑ 10.95)	Inflammatory response<br>Transcription factor<br>Complementary response<br>Complementary response<br>Complementary response<br>Complementary response<br>Serine proteinase and complement inhibitor<br>Serine proteinase inhibitor	A2m（↑, 4.45)<br>Cebpd (↑, 5.11)<br>C1qa (↑, 6.54)<br>C1qb (↑, 2.5)<br>C1qc (↑, 2.14)<br>C4 (↑, 8.88)<br>Serping1 (↑, 16.64)<br><br>Serpina3n（↑, 6.5)	[3]
Obesity-associated retinal degeneration	WNIN-Ob rat	Slc31a2 (↓, 0.20)<br>RGD1308116 (↓, 0.08)<br>Hk2 (↓, 0.07)<br><br>Pde6a (↓, 0,08)	copper ion transport<br>protein phosphorylation<br>glucose metabolic process and hexose transport<br>visual transduction	Slc31a2 (↓, 0.07)<br>RGD1308116 (↓, 0.08)<br>Hk2 (↓, 0.08)<br><br>Pde6a (↓, 0.31)	[4]

		Pde6b (↓, 0.16)<br>Rom1 (↓, 0.23)<br><br>Lgals3 (↑, 6.27)<br>Ctss (↑,3.73)<br>Serping1 (↑, 3.39)<br><br>C1qa (↑, 3.22)<br>Cp（↑, 3.07)	visual transduction<br>response to stimulus and visual perception<br>Adhesion and immune response<br>Antigen processing and presentation<br>Serine proteinase and complement inhibitor<br>Complementary response<br>Ferroxidase and iron homestasis	Pde6b (↓, 0.43)<br>Rom1 (↓, 0.39)<br><br>Lgals3 (↑, 18.99)<br>Ctss (↑, 4.93)<br>Serping1 (↑, 16.64)<br><br>C1qa (↑, 6.54)<br>Cp（↑, 6.39)	
X-linked juvenile retinoschisis (RS)	*Rs1h* knockout mice	Spp1 (↑, 4.3)<br>Cd68 (↑, 2.1)<br>Cebpd (↑, 2.1)<br>Lgals3 (↑, 1.9)<br>C1qb (↑, 1.8)	Cell adhesion and immune response<br>Macrophage protein<br>Transcription factor<br>Adhesion and immune response<br>Complementary response	Spp1 (↑, 3.83)<br><br>Cebpd (↑, 5.11)<br>Lgals3 (↑, 18.99)<br>C1qb (↑, 2.5)	[5]
Retinal light damage	Light damage model of retinal degeneration in Balb/c mice	Cp（↑, 5.5)<br>B2m（↑, 2.7)<br>Cebpd (↑, 11.1)<br>Stat1（↑, 3.9)<br>A2m（↑, 3.8)<br>Spp1（↑, 5.1)<br>Serpina3n（↑, 3.5)	Ferroxidase and iron homestasis<br>Antigen processing and presentation<br>Transcription factors<br>Transcription factors<br>Inflammatory response<br>Cell adhesion and immune response<br>Serine proteinase inhibitor	Cp（↑, 6.39)<br>B2m（↑, 4.3)<br>Cebpd (↑, 5.11)<br>Stat1（↑, 3.91)<br>A2m（↑, 4.45)<br>Spp1（↑, 3.83)<br>Serpina3n（↑, 6.5)	[6]
Glaucoma	Experimental elevation of intraocular pressure (IOP) in a rat (Brown	Cd74 (↑, 19.6)<br>RT1-DA（↑, 8.4)<br>B2m（↑, 2.6)<br>C1qb (↑, 15.7)<br>C1s (↑, 4.7)	Antigen processing and presentation<br>Antigen processing and presentation<br>Antigen processing and presentation<br>Complementary response<br>Complementary response	Cd74 (↑, 21.64)<br>RT1-DA（↑, 6.22)<br>B2m（↑, 4.3)<br>C1qb (↑, 2.5)<br>C1s (↑, 9.75)	[7]

	Norway rats) glaucoma model	C1r (↑, 3.5)<br>C3 (↑, 10.0)<br>A2m (↑, 9.2)<br>Lgals3 (↑, 7.9)<br>Timp1 (↑, 45.8)<br>Serpina3n (↑, 9.2)<br>Serping1 (↑, 3.8)<br>Cebpd (↑, 8.1)<br>Cp (↑, 5.9)	Complementary response<br>Complementary response<br>Inflammatory response<br>Adhesion and immune response<br>Protease inhibitor<br>Serine proteinase inhibitor<br>Serine proteinase and complement inhibitor<br>Transcription factor<br>Ferroxidase and iron homestasis	C1r (↑, 2.33)<br>C3 (↑, 9.39)<br>A2m (↑, 4.45)<br>Lgals3 (↑, 18.99)<br>Timp1 (↑, 8.33)<br>Serpina3n (↑, 6.5)<br>Serping1 (↑, 16.64)<br>Cebpd (↑, 5.11)<br>Cp (↑, 6.39)	
Hyperoxia	C57BL/6J mice exposed to hyperoxia	C3 (↑, 10.41)<br>C4 (↑, 5.41)<br>C1qa (↑, 2.37)<br>C1qb (↑, 4.56)<br>C1qc (↑, 6.43)<br>Serping1 (↑, 5.26)	Complementary response<br>Complementary response<br>Complementary response<br>Complementary response<br>Complementary response<br>Serine proteinase and complement inhibitor	C3 (↑, 9.39)<br>C4 (↑, 8.88)<br>C1qa (↑, 6.54)<br>C1qb (↑, 2.5)<br>C1qc (↑, 2.14)<br>Serping1 (↑, 16.64)	[8]
Diabetes	Retinal Müller cells of STZ-induced diabetic rats (SD rats)	Cd74 (↑, 3.3)<br>Spp1 (↑, 9.8)<br>C1s (↑, 10.1)<br>C3 (↑, 4.6)<br>Serping1 (↑, 8.9)<br>Cp (↑, 3.1)<br>A2m (↑, 2.8)<br>Serpina3n (↑, 15.3)	Antigen processing and presentation<br>Cell adhesion and immune response<br>Complementary response<br>Complementary response<br>Serine proteinase and complement inhibitor<br>Ferroxidase and iron homestasis<br>Inflammatory response<br>Serine proteinase inhibitor	Cd74 (↑, 21.64)<br>Spp1 (↑, 3.83)<br>C1s (↑, 9.75)<br>C3 (↑, 9.39)<br>Serping1 (↑, 16.64)<br>Cp (↑, 6.39)<br>A2m (↑, 4.45)<br>Serpina3n (↑, 6.5)	[9]

	STZ-induced diabetic rats (SD rats)	Timp1（↑, 15.2)<br>Cebpd (↑, 7.9)<br>Serping1 (↑)<br><br>Lgals3 (↑)<br>Timp1（↑)	Protease inhibitor<br>Transcription factor<br>Serine proteinase and complement inhibitor<br>Adhesion and immune response<br>Protease inhibitor	Timp1（↑, 8.33)<br>Cebpd (↑, 5.11)<br>Serping1 (↑, 16.64)<br><br>Lgals3 (↑, 18.99)<br>Timp1（↑, 8.33)	[10]

1. Demos C, Bandyopadhyay M, Rohrer B. (2008) Identification of candidate genes for human retinal degeneration loci using differentially expressed genes from mouse photoreceptor dystrophy models. Mol Vis. 14:1639-49
2. Genini S**,** Zangerl B**,** Slavik J.(2010) Transcriptional profile analysis of RPGRORF15 frameshift mutation identifies novel genes associated with retinal degeneration. Invest Ophthalmol Vis Sci. 51:6038-50
3. Schaeferhoff K, Michalakis S, Tanimoto N. (2010) Induction of STAT3-related genes in fast degenerating cone photoreceptors of cpfl1 mice. Cell Mol Life Sci. 67:3173-86
4. Reddy GB, Vasireddy V, Mandal MN. (2009) A novel rat model with obesity-associated retinal degeneration. Invest Ophthalmol Vis Sci.50:3456-63
5. Gehrig, A., Langmann, T., Horling, F., et al. (2007) Genome-wide expression profiling of the retinoschisin-deficient retina in early postnatal mouse development. Invest. Ophthalmol. Vis. Sci. 48**:**891–900
6. Chen, L., Wu, W., Dentchev, T.,et al. (2004) Light damage induced changes in mouse retinal gene expression. Exp. Eye Res. 79**:** 239 –247
7. Ahmed, F., Brown, K. M., Stephan, D. A., et al. (2004) Microarray analysis of changes in mRNA levels in the rat retina after experimental elevation of intraocular pressure. Invest. Ophthalmol. Vis. Sci. 45: 1247–1258
8. Natoli R, Provis J, Valter K, et al. (2008) Gene regulation induced in the C57BL/6J mouse retina by hyperoxia: a temporal microarray study. Molecular Vision 14:1983-1994
9. Gerhardinger C, Costa MB, Coulombe M, et al.(2005) Expression of acute-phase response proteins in retinal Müller cells in diabetes. Invest Ophthalmol Vis Sci. 46:349–357
10. Brucklacher RM, Patel KM, VanGuilder HD, et al. (2008) Whole genome assessment of the retinal response to diabetes reveals a progressive neurovascular inflammatory response. BMC Med Genomics. 1:26
